# HYPHYCA: a prospective study in 613 patients conducting a comprehensive analysis for predictive factors of physiological ^18^F-FDG anal uptake

**DOI:** 10.1186/s13550-020-0615-5

**Published:** 2020-03-20

**Authors:** Nicolas Aide, Laure-Eugénie Tainturier, Cathy Nganoa, Benjamin Houdu, Jennifer Kammerer, Marie-Pierre Galais, Renaud Ciappuccini, Charline Lasnon

**Affiliations:** 1 Normandie University, Caen, France; 2 Nuclear Medicine Department, Caen University Hospital, Avenue Côte de Nacre, 14000 Caen, France; 3 INSERM 1086 ANTICIPE, Normandie University, Caen, France; 4 Radiation Oncology Department, François Baclesse Cancer Centre, Caen, France; 5 Digestive Oncology Department, François Baclesse Cancer Centre, Caen, France; 6 Nuclear Medicine Department, François Baclesse Cancer Centre, Caen, France

**Keywords:** PET, ^18^F-FDG, Anal cancer, Physiological uptake

## Abstract

**Background:**

Anal cancer is a relatively rare tumor of which incidence increases in developed countries. ^18^F-FDG PET has been increasingly used for its post radio-chemotherapy evaluation. However, several authors have reported the risk of local false-positive findings leading to low specificity and positive predictive values. These false-positive results could be due to post-radiotherapy inflammation or infection but certainly also to physiological anal canal uptake that is observed on a regular basis in clinical practice. The purpose of this prospective study (NCT03506529; HYPHYCA) was therefore to seek predictive factors of physiological anal canal hypermetabolism.

**Materials and methods:**

Over a 2-month period, patients aged 18 years old and more, referred for ^18^F-FDG PET-CT at two EARL-accredited PET centers were included, after obtaining their informed and written consent. They were asked to fill in a questionnaire including seven closed questions about usual intestinal transit, ongoing medications relative to intestinal transit, history of digestive, and anal and/or pelvic diseases. Age, gender, and body mass index (BMI) were recorded. A single nuclear medicine physician visually and quantitatively analyzed anal canal uptake (SUV_max_EARL_) and assessed visual rectal content (air, feces, or both) and the largest rectal diameter (mm).

**Results:**

Six hundred and thirteen patients were included (sex ratio F/M = 0.99) and 545 (89%) questionnaires were entirely completed. Significantly more males presented anal canal hypermetabolism (sex ratio (M/F) = 1.18 versus 0.85, *p* = 0.048). Moreover, patients with anal canal hypermetabolism had higher BMI (27.6 (5.7) kg/m^2^ versus 23.9 (4.5) kg/m^2^, *p* < 0.0001), higher rate of hemorrhoid history (43% versus 27%, *p* = 0.016), and higher rate of rectum filled with only feces (21% versus 12%, *p* = 0.019) as compared to patients with no anal canal uptake. On logistic regression, all these variables were found to be independent predictors of the occurrence of an anal canal hypermetabolism. Odds ratio were 1.16 (1.12–1.20) per unit of BMI (kg/m^2^) (*p* < 0.0001), 1.48 (1.04–2.11) for males (*p* = 0.030), 1.64 (1.10–2.45) for hemorrhoids history (*p* = 0.016), and 1.94 (1.147–3.22) for the rectum filled with only feces (*p* = 0.010).

**Conclusion:**

According to our study, the predictive factors of physiological anal canal hypermetabolism are high BMI, male gender, hemorrhoid history, and rectum filled with only feces. This may pave the way to a more specific interpretation of post radio-chemotherapy PET evaluations of anal canal cancer, provided that other studies are conducted in this specific population.

**Trial registration:**

This prospective study was registered at Clinicaltrial.gov: NCT03506529; HYPHYCA on April 24, 2018

## Background

Anal cancer is a relatively rare tumor with incidence rates between 1.0 and 2.0 per 100,000 people a year in most Western countries. However, its incidence increases by 2 to 3% per year in developed countries, especially among the youngest male homosexuals and immunocompromised persons [1]. Indeed, the known risk factors for anal cancer are infections by human papillomavirus, human immunodeficiency virus, sexual risk factors (homosexuality in men and multiple sexual partners in women), and tobacco exposure. Traditional staging techniques based on surgical and anatomo-pathological parameters have been supplanted by clinical staging because external radiotherapy and chemotherapy (5-fluorouracile and mitomycin C) have replaced surgery as the main treatment of choice [2]. ^18^F-Fluorodeoxyglucose (^18^F-FDG) positron emission tomography (PET) has been increasingly evaluated in the management of anal cancer patients, particularly for the initial staging of the disease and the evaluation of the therapeutic response after radiochemotherapy. In this context of post-treatment evaluation, thanks to its good negative predictive value with reported values superior to 90%, ^18^F-FDG PET can avoid biopsies in case of a complete metabolic response [3–5]. This is clinically relevant as post radiotherapy biopsies can lead to necrosis and potentially complicated and delayed surgery. However, several authors reported the risk of local false-positive PET [6, 7], leading to low specificity and positive predictive values [4]. For example, in the Nguyen et al. study [7], 5 patients were classified in partial metabolic response, but after biopsy, 3 of them were in fact in complete local response. Similar results were found by Vercellino et al. with a ^18^F-FDG PET/CT specificity of only 81% [5]. These false-positive results could result not only from post-radiotherapy inflammation or infection, but also from physiological anal canal uptake that is frequently observed in daily clinical practice and is therefore a critical parameter to be taken into account.

To our knowledge, there is currently no large prospective study exploring the predictive factors of physiological anal hypermetabolism. The purpose of this study was therefore to seek the factors of physiological anal canal hypermetabolism in the general PET population in an attempt to pave the way to the improvement of interpretation in anal cancer patients by reducing the risk of false-positive PET results and therefore improving specificity and positive predictive values.

## Materials and methods

### Population and questionnaire

This study was a prospective bicentric observational study. All patients aged of 18 years old and more, referred for a ^18^F-FDG PET-CT at the Centre François Baclesse and the Caen University Hospital PET centers, were included after their informed and written consent was obtained. The inclusion period took place from September 17, 2018 to November 15, 2018. Of note, preliminary results of this study were presented during the 2019 EANM congress [8].

We excluded all patients with a history of anal cancer, biguanides-induced colitis on PET images, or colostomy and all patients deprived of liberty, under tutorship or curatorship, or with any associated socio-educational, medical, or psychological condition that could compromise their ability to participate in the study (e.g., illiteracy and mental disability). All procedures in this study involving human participants were performed in accordance with the ethical standards of the institutional or national research committee and with the principles of the 1964 Declaration oh Helsinki and its later amendments or comparable ethical standards. The study was registered in the Clinical Trials Protocol Registration System (NCT03506529; HYPHYCA).

All enrolled patients were asked to fill in the questionnaire during the ^18^F-FDG uptake time. The questionnaire used was designed specifically for our study in consensus between PET readers from our two PET centers and radiation oncologists and a gastroenterologist from our digestive tumor board. The time required for filling in the questionnaire was estimated at about 10 min. It included seven closed questions concerning the usual intestinal transit, ongoing medications relative to intestinal transit, and history of digestive, anal, and/or pelvic treatments (Table 1). A specific identification number was then assigned to each patient, and both the surveys and PET data were anonymized for further analysis. The medical care of the patients in PET units was not modified in any way. No additional examination was carried out and no follow-up of patients was necessary in this study. In addition to the questionnaire, age, gender, body mass index (BMI), and PET indication were recorded.

**Table 1:** Patients characteristics

Patients characteristics	Total population (*n* = 613)	Hypermetabolism (*n* = 318)	Basal metabolism (*n* = 295)	*p* value
**Age (years)**				
Mean ± SD	61 ± 14	62 ± 13	61 ± 15	0.518
**BMI (kg/m**^**2**^**)**				
Mean ± SD	25.9 ± 5.4	27.7 ± 5.5	24.0 ± 4.4	**< 0.0001**
**Gender (*n*, %)**				
Females	305 (49.8)	146 (45.9)	159 (53.9)	**0.048**
Males	308 (50.2)	172 (54.1)	136 (46.1)	

### PET/CT-acquisition and reconstruction

Patients’ preparation in the PET unit and PET acquisition and reconstructions were performed as per the EANM guidelines for PET tumor imaging [9], and both PET centers are EARL accredited [10]. Injected dose, time between injection and acquisition, and capillary glycemia were recorded. PET/CT acquisitions were performed using two different PET/CT scanners: a Biograph TrueV with extended field-of-view (Siemens Medical Solutions) and a Vereos system (Philips Medical Systems). Patients were asked to fast for 6 h or more before ^18^F-FDG injection. Patients’ weight was checked on a calibrated scale [11]. PET/CT images were performed 60 min post injection from mid-thigh to the base of the skull except for lower limb melanoma and myeloma where whole-body acquisitions were performed.

Concerning the Biograph TrueV system, the injected dose was 4.0 MBq/kg and the acquisition time per bed position was set to 2 min and 40 s for patients with BMI < 25 kg/m^2^ and 3 min and 40 s for patients with BMI ≥ 25 kg/m^2^. To fulfill the EARL accreditation, PET raw data were reconstructed with a 3D-OSEM reconstruction algorithm with point spread function (PSF) modeling (3 iterations and 21 subsets) and a 6.3-mm post-reconstruction Gaussian filter, using a 128 × 128 matrix size. Scatter and attenuation corrections were applied.

For the Vereos system, the injected dose was 3.0 MBq/kg and the acquisition time per bed position was set to 2 min whatever the patients’ body habitus. To fulfill the EARL accreditation, PET raw data were reconstructed with a 3D-OSEM reconstruction algorithm with PSF modeling (2 iterations and 10 subsets) with a 5-mm post-reconstruction Gaussian filter, using a 288 × 288 matrix size. Scatter and attenuation corrections were applied.

### PET/CT analysis

A nuclear medicine physician reviewed all PET images on a digital workstation (ISP software, Philips). For all examinations, EARL-compliant quantitative analysis was used [10]. The following features were recorded for all examinations:

- Visual analysis of the hypermetabolism of the anal canal using a 3-point scale (0, no hypermetabolism; 1, moderate hypermetabolism; 2, intense hypermetabolism).
- Anal canal maximum standardized uptake values (SUV_max___EARL_). VOI was determined using 50% of SUV_peak_ with adaptation to local tumor-to-background contrast, so called adapted 50% of SUV_peak_ (A50P) [12].
- Visual assessment of rectal content using a 4-point scale (empty; 0, air; 1, feces; 2, air and feces).
- Largest rectal diameter (mm).

To do an inter-observers’ agreement analysis for the discrimination between patients with a basal anal canal hypermetabolism (classified 0) and those with a significant anal canal hypermetabolism (classified 1 or 2), a second nuclear medicine physician was randomly assigned 100 PET/CT examinations. He recorded visual analysis of the hypermetabolism of the anal canal using a 3-point scale and the anal canal SUV_max___EARL_ as described above.

### Statistical analysis

Continuous quantitative data are presented as mean ± SD. Categorical data are presented as frequencies and percentages. Mean SUV_max_ values between patients with visually basal anal canal metabolism (0), moderate hypermetabolism (1), and intense hypermetabolism (2) were compared using a Kruskal-Wallis test. Quantitative data of patients with basal anal canal metabolism (0) and patients with a hypermetabolism (1 and 2) were compared using Mann-Whitney tests. Categorical data of patients with a basal anal canal metabolism (0) and patients with a hypermetabolism (1 and 2) were compared using chi-squared tests. When appropriate, linear regression was used to seek an association between quantitative variables and ROC analysis was used to determine optimal cutoff values based on the Youden index. Inter-observers’ agreement was assessed by the use of a Cohen’s kappa coefficient. To disentangle the effects of several variables on the occurrence of an anal canal hypermetabolism, a logit logistic regression was performed taking into account all significant variables on univariable analysis.

## Results

### Population characteristics

**Fig. 1:**
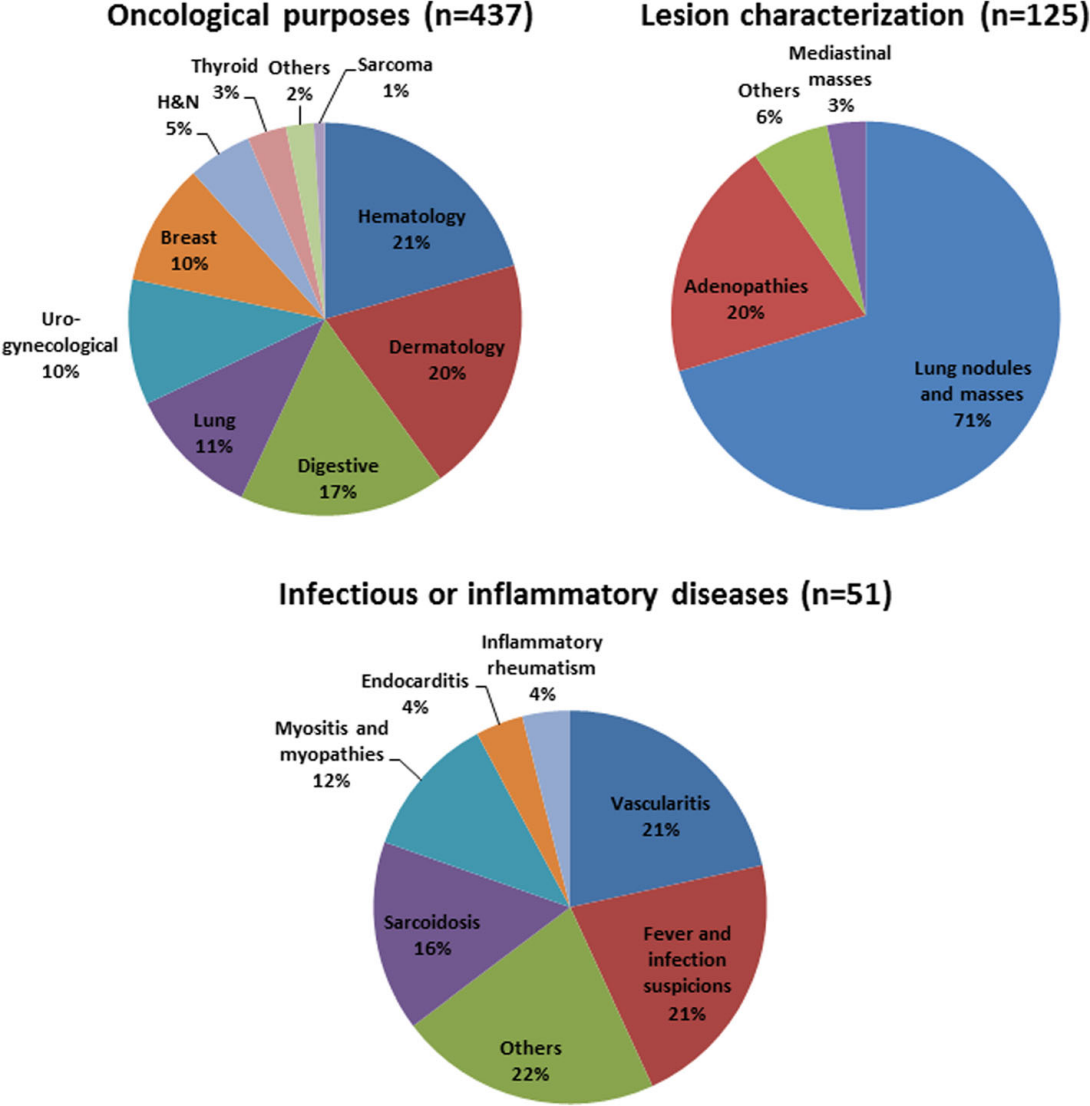
Additional details on PET examinations indications

**Fig. 2:**
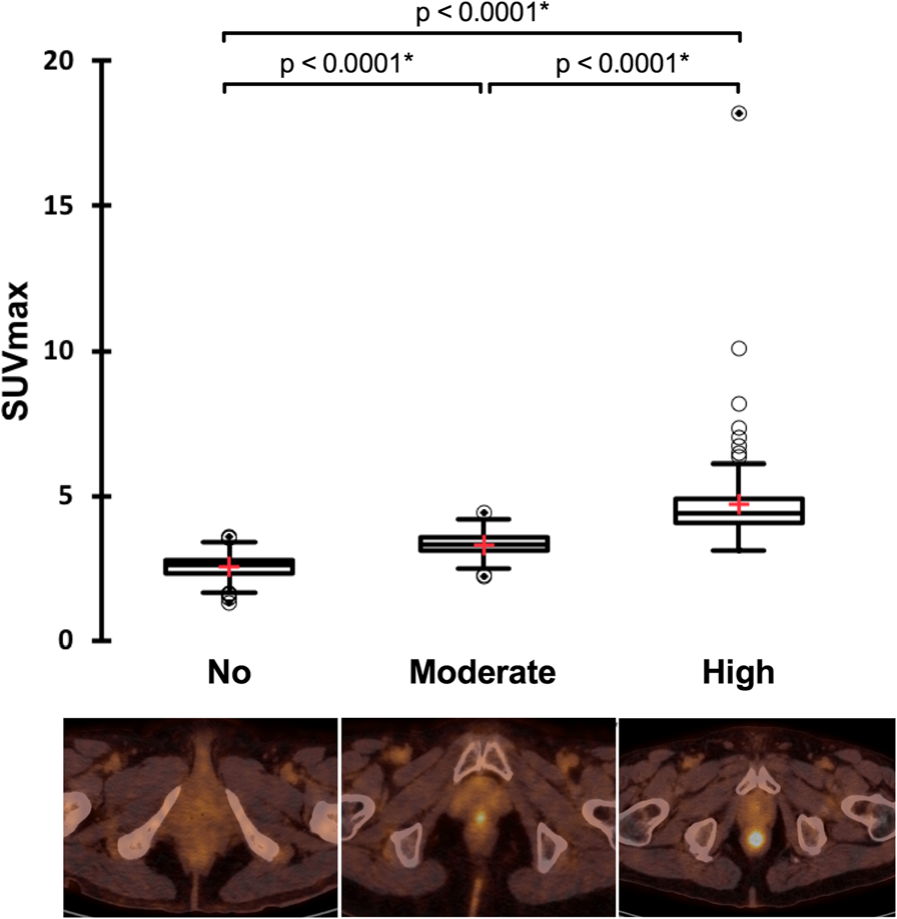
Visual versus quantitative analysis of anal canal uptake. Data are presented as Tukey’s boxplots and o represents outliers

**Fig. 3:**
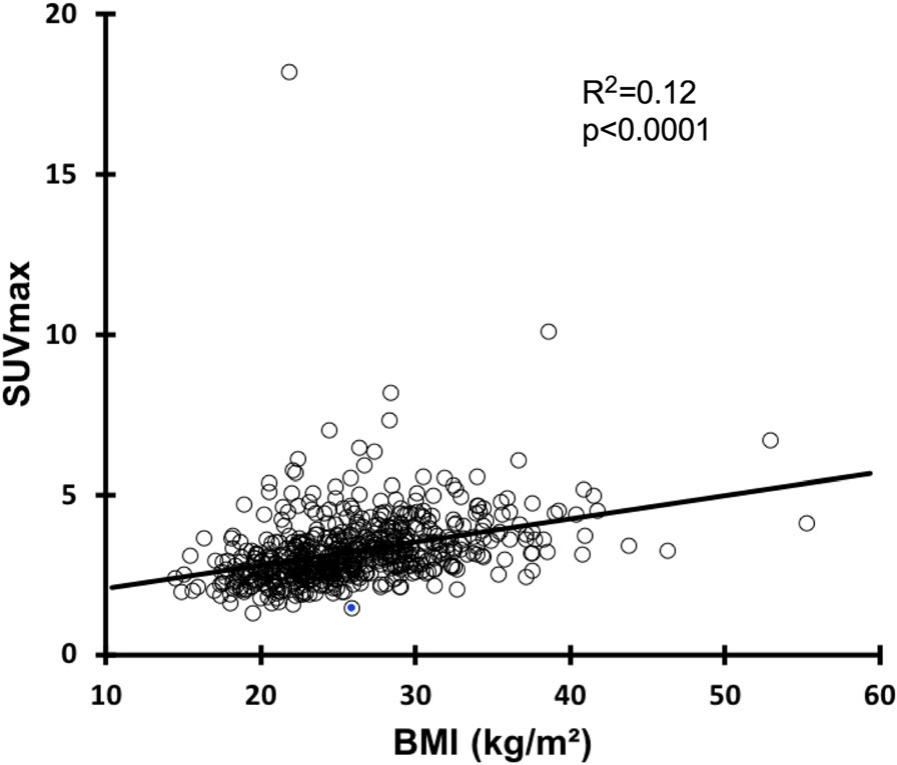
Linear regression of SUV_max_EARL_ and body mass index (kg/m^2^)

Six hundred and thirty-nine patients were initially included. Twenty-six were excluded (19 diffuse colon uptake on PET images, 3 histories of anal cancers, 4 colostomies) resulting in a final database of 613 patients (305 females and 308 males). Diffuse colon uptake on PET images was due to ulcerative colitis (*n* = 1), biguanides (*n* = 8), immunotherapy (*n* = 3), or unknown etiologies (*n* = 7). Patient’s characteristics are displayed in Table 1. Four hundred and thirty-seven patients were referred for oncological purposes, 51 for infectious or inflammatory diseases, and 125 to characterize a lesion whatever the site concerned. For more details, refer to Fig. 1. Two hundred and ninety-five patients had basal anal canal metabolism (classified 0), 193 patients presented a moderate anal canal hypermetabolism (classified 1) and 125 with an intense anal canal hypermetabolism (classified 2). Corresponding mean SUV_max_ values were equal to 2.56 ± 0.39, 3.32 ± 0.35, and 4.72 ± 1.54, respectively (Fig. 2, *p* < 0.0001). ROC curves analyses showed that the optimal SUV_max___EARL_ cutoff value to discriminate between patients with a basal anal canal metabolism (0) and patients with a hypermetabolism (1 and 2) was > 2.96 (AUC = 0.955, *p* < 0.0001, accuracy = 90.0%). Of note, the optimal SUV_max___EARL_ cutoff value to discriminate between patients with low and high anal canal uptake hypermetabolism was ≥ 3.78 (AUC = 0.962, *p* < 0.0001, accuracy = 90.6%). There were significant differences in BMI and gender between patients with basal anal canal metabolism and patients presenting an anal canal hypermetabolism with significantly higher BMI (27.6 ± 5.7 kg/m^2^ versus 23.9 ± 4.5 kg/m^2^, *p* < 0.0001) and significantly more males (sex ratio (M/F) = 1.18 versus 0.85, *p* = 0.048) in the group presenting anal canal hypermetabolism (Table 1). There was a significant linear correlation between BMI and anal canal SUV_max_EARL_ values with *R*^2^ = 0.12 (*p* < 0.0001, Fig. 3). ROC curves analysis showed that the optimal cutoff to discriminate between patients with and without anal canal hypermetabolism was a BMI ≥ 26.22 (AUC = 0.705, *p* < 0.0001). Mean age was not significantly different between these two groups of patients (Table 1).

### Questionnaire analysis

Five hundred and forty-five (89%) questionnaires were entirely completed. Numbers of completed questionnaires on a per question basis as well as corresponding responses are detailed in Table 2. Patients presenting an anal canal hypermetabolism had a significantly higher rate of hemorrhoid histories as compared to patients with basal anal canal metabolism: 43% versus 27%, respectively (*p* = 0.016). There were no differences in the number of stools per day, delay between last stool and FDG injection, Bristol stool form scale [13] ongoing treatments, anal fissure or abscess histories, surgery or radiotherapy histories, inflammatory bowel disease history, and anal invasive act history between these two groups of patients (Table 2). Concerning patients with surgery history (*n* = 131), time data were not available for 13 patients (9.9%). Nineteen patients (14.5%) underwent anal, rectal, or pelvic surgery during the year before the PET examination. This group of patients did not display significantly different anal canal metabolism compared to the rest of the population: 3.25 ± 0.99 versus 3.22 ± 1.1, respectively (*p* = 0.83). Concerning patients with anal, rectal, or pelvic irradiation history (*n* = 49), time data were not available for 5 patients (10.2%). Thirteen patients (26.5%) underwent anal, rectal, or pelvic surgery during the 3 months before the PET examination. This group of patients did not display significantly different anal canal metabolism compared to the rest of the population: 3.50 ± 0.77 versus 3.22 ± 1.12, respectively (*p* = 0.11).

**Table 2:** Questionnaires data

Questions	Total population	Hypermetabolism (n= 318)	Basal metabolism (n= 295)	p value
**1. Number of stools per day (n, %)**				
Number of answers	605	314	291	
0	117 (19.3)	63 (20.1)	54 (18.6)	0.869
1	309 (51.1)	157 (50.0)	152 (52.2)	
2	131 (21.7)	67 (21.3)	64 (22.0)	
3	48 (7.9)	27 (8.6)	21 (7.2)	
**2. Delay between last stool and FDG injection (hours)**				
Number of answers	590	306	84	
Mean (SD)	14.9 (16.7)	14.5 (16.6)	15.3 (16.8)	0.238
**3. Bristol classification (**n**, %)**				
Number of answers	586	306	280	
0, constipation (1–2)	114 (19.5)	58 (18.9)	56 (20.0)	0.949
1, normal (3–4)	346 (59.0)	182 (59.5)	164 (58.6)	
2, diarrhea (5–7)	126 (21.5)	66 (21.6)	60 (21.4)	
**4. Ongoing treatments**				
**Laxative treatment (n, %)**				
Number of answers	603	312	291	
Yes	57 (9.5)	27 (8.7)	30 (10.3)	0.487
No	546 (90.5)	285 (91.3)	261 (89.7)	
**Anti-diarrhetic treatment (n, %)**				
Number of answers	601	311	290	
Yes	27 (4.5)	17 (5.5)	10 (3.4)	0.233
No	574 (95.5)	294 (94.5)	280 (96.6)	
**5. Anal, rectal, and pelvic history**				
**Hemorrhoid history (n, %)**				
Number of answers	598	309	289	
Yes	155 (25.9)	93 (30.1)	62 (21.5)	**0.016**
No	443 (74.1)	216 (69.9)	227 (78.5)	
**Anal fissure history (n, %)**				
Number of answers	600	310	290	
Yes	20 (3.3)	12 (3.9)	8 (2.8)	0.448
No	580 (96.7)	298 (96.1)	282 (97.2)	
**Anal abscess history (n, %)**				
Number of answers	599	310	289	
Yes	4 (0.7)	2 (0.6)	2 (0.7)	0.944
No	595 (99.3)	308 (99.4)	287 (99.3)	
**Anal, rectal, or pelvic surgery history (n, %)**				
Number of answers	602	312	290	
Yes	131 (21.8)	67 (21.5)	64 (22.1)	0.860
No	471 (78.2)	245 (78.5)	226 (77.9)	
**Anal, rectal, or pelvic irradiation history (n, %)**				
Number of answers	602	311	291	
Yes	49 (8.1)	27 (8.7)	22 (7.6)	0.615
No	553 (91.9)	284 (91.3)	269 (92.4)	
**6. Inflammatory bowel disease history (n, %)**				
Number of answers	603	312	291	
Yes	12 (2.0)	8 (2.6)	4 (1.4)	0.296
No	591 (98.0)	304 (97.4)	287 (98.6)	
7. Anal invasive act history* (n, %)				
Number of answers	603	312	291	
Yes	55 (9.1)	30 (9.6)	25 (8.6)	0.662
No	548 (90.9)	282 (90.4)	266 (91.4)	

*All types of anal invasive examinations (rectal enema, rectoscopy, coloscopy, etc.) or anal intercourse

### PET/CT characteristics

The mean injected dose of ^18^F-FDG was equal to 3.47 ± 0.56 MBq/kg, the mean blood glucose level was equal to 1.02 ± 0.21 g/l, and the mean uptake period was equal to 59 ± 4 min. There were no significant differences in the injected dose, blood glucose level, and uptake period between patients with basal anal canal metabolism and those presenting an anal canal hypermetabolism (Table 3).

**Table 3:** PET/CT characteristics

PET/CT characteristics	Total population (*n* = 613)	Hypermetabolism (*n* = 318)	Basal metabolism (*n* = 295)	*p* value
**Mean injected dose (MBq/kg)**				
Mean ± SD	3.47 ± 0.56	3.44 ± 0.56	3.50 ± 0.57	0.454
**Mean blood glucose level (g/L)**				
Mean ± SD	1.02 ± 0.21	1.03 ± 0.22	1.02 ± 0.21	0.996
**Mean**^**18**^**F-FDG uptake time (min)**				
Mean ± SD	59 ± 4	59 ± 5	59 ± 4	0.697
**Rectal diameter (cm)**				
Mean ± SD	41 ± 15	42 ± 14	40 ± 15	0.202
**Rectal content (*n*, %)**				
Empty	84 (13.7)	37 (11.6)	47 (15.9)	**0.019**
0, air	270 (44.0)	134 (42.1)	136 (46.1)	
1, feces	101 (16.5)	66 (20.8)	35 (11.9)	
2, air and feces	158 (25.8)	81 (25.5)	77 (26.1)	

**Fig. 4:**
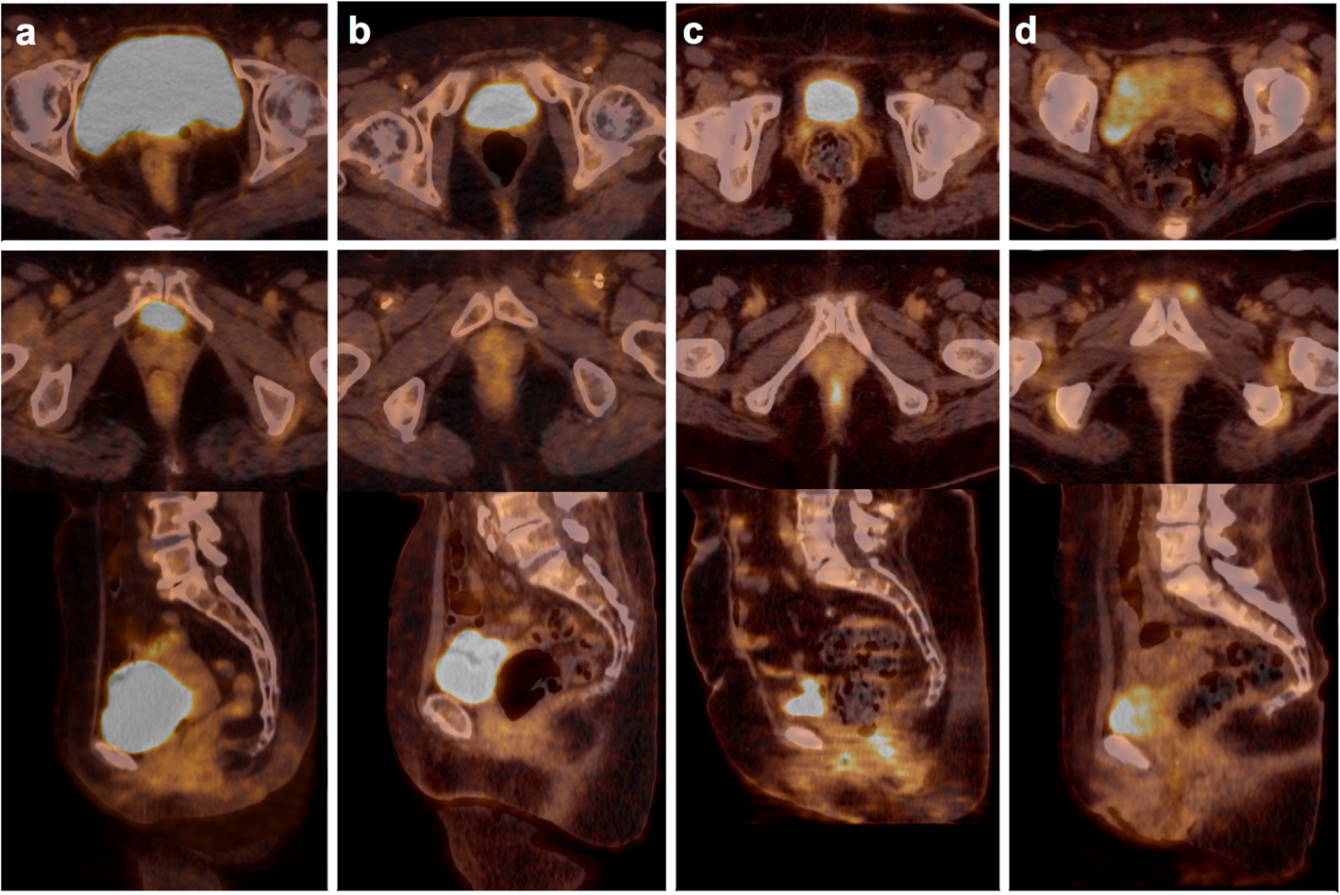
Illustration of rectal content classification and its association with anal canal uptake. **a** Empty rectum. **b** Air rectal content classified 0. **c** Fecal rectal content classified 1. **d** Mixed rectal content classified 2

Concerning CT characteristics, there was a significantly higher rate of rectum filled with only feces (scale 1) in patients presenting with an anal canal hypermetabolism as compared to those with basal anal canal metabolism: 21% versus 12%, respectively (*p* = 0.019, Table 3, Fig. 4). No difference was found in the rectal diameter between these two groups of patients (Table 3).

### Logistic regression

The logistic regression took into account all significant variables on univariable analysis, that is to say BMI, gender, hemorrhoids history, and rectal content. The analysis was performed on the 598 patients for whom all these variables were recorded. All variables were found to be independent predictors of the occurrence of an anal canal hypermetabolism (Table 4). Odds ratio were 1.16 (1.12–1.20) per unit of BMI (kg/m^2^) (*p* < 0.0001), 1.48 (1.04–2.11) for males (*p* = 0.030), 1.64 (1.10–2.45) for hemorrhoids history (*p* = 0.016), and 1.94 (1.147–3.22) for the rectal content type 1 (only feces) (*p* = 0.010).

**Table 4:** Logistic regression analysis of risk factors for anal canal hypermetabolism

Parameter	Value	Standard error	Wald chi-square	Pr > chi^**2**^
**Intercept**	− 4.340	0.544	63.547	**< 0.0001**
**BMI (kg/m**^**2**^**)**	0.155	0.020	60.051	**< 0.0001**
**Gender—male**	0.392	0.181	4.688	**0.030**
**Hemorrhoid history**	0.493	0.205	5.790	**0.016**
**Rectal content --1 (feces)**	0.666	0.259	6.587	**0.010**
**Rectal content— 2 (air and feces)**	− 0.043	0.220	0.038	0.846
**Rectal content— empty**	0.039	0.278	0.020	0.888

### Inter-observers’ agreement assessment on a random dataset of 100 patients

Using visual analysis to discriminate between patients with a basal anal canal metabolism (0) and patients with a hypermetabolism (1 and 2), the agreement between the two observers was strong with a Cohen’s kappa coefficient equal to 0.64. There were discrepancies in 18 patients. A linear regression showed an almost perfect correlation between SUV_max___EARL_ values extracted by the two observers with a *R*^2^ equal to 0.95 (*p* < 0.0001). The agreement between observers was noticeably improved when using SUV_max___EARL_ quantitative data and applying the previously described cutoff value of 2.96. Using this method, the Cohen’s kappa coefficient was found to be almost perfect, equal to 0.88, with only 6 discrepancies.

## Discussion

According to our study, the predictive factors of physiological anal canal hypermetabolism are male gender, high BMI, hemorrhoids history, and rectum filled with only feces.

Surprisingly, concerning patient characteristics, men displayed significantly more anal canal hypermetabolism than women. This could be potentially explained by anorectal manometric studies that showed that anal sphincters were significantly longer in men than women and that mean squeeze pressure was higher in men [14]. For these reasons, it could be plausible that anal canal metabolism could be better seen in male than in female patients. Concerning body habitus, mean BMI was higher in patients presenting canal anal uptake. Furthermore, ROC curves analysis seems to indicate that overweight and obese patients (BMI ≥ 26.22) are more likely to present an anal canal hypermetabolism which is actually easy to take into account in our routine practice, even though the background of this finding is not yet clarified.

The interrogation of the patient in the search for a history of hemorrhoids appears to be a crucial issue, whatever the date of the last hemorrhoidal surge. Indeed, in our study, stratification according to the time interval of the last hemorrhoidal surge was not necessary to obtain statistical significance. This antecedent should be systematically sought before any post-treatment PET evaluation of anal cancer, especially as it is the most frequently encountered affection in proctology [15] and as radiotherapy is a known provider of hemorrhoidal crisis [16].

Finally, we found the rectum filled with only feces to be a predictor of anal canal hypermetabolism as compared to other rectal content. From a functional point of view, the anal canal is above all a sphincteric apparatus. The arrival of materials in the rectal ampoule physiologically leads to an associative sampling reflex associating a propulsive rectal contraction, a relaxation of the internal anal sphincter and a reflex contraction of the external anal sphincter. The subsequent stage, continence or defecation, is under the control of the will of the individual who chooses to respond or not to the exonerating need [17]. Therefore, we can hypothesize that the anal canal uptake could be linked to the voluntary solicitation of the external anal sphincter at the time of the FDG injection and/or during the uptake FDG period. To avoid this phenomenon, we could quite easily consider the use of a rectal enema in the hours preceding the post-treatment PET evaluation as it is already done during the radiotherapy before each session.

**Fig. 5:**
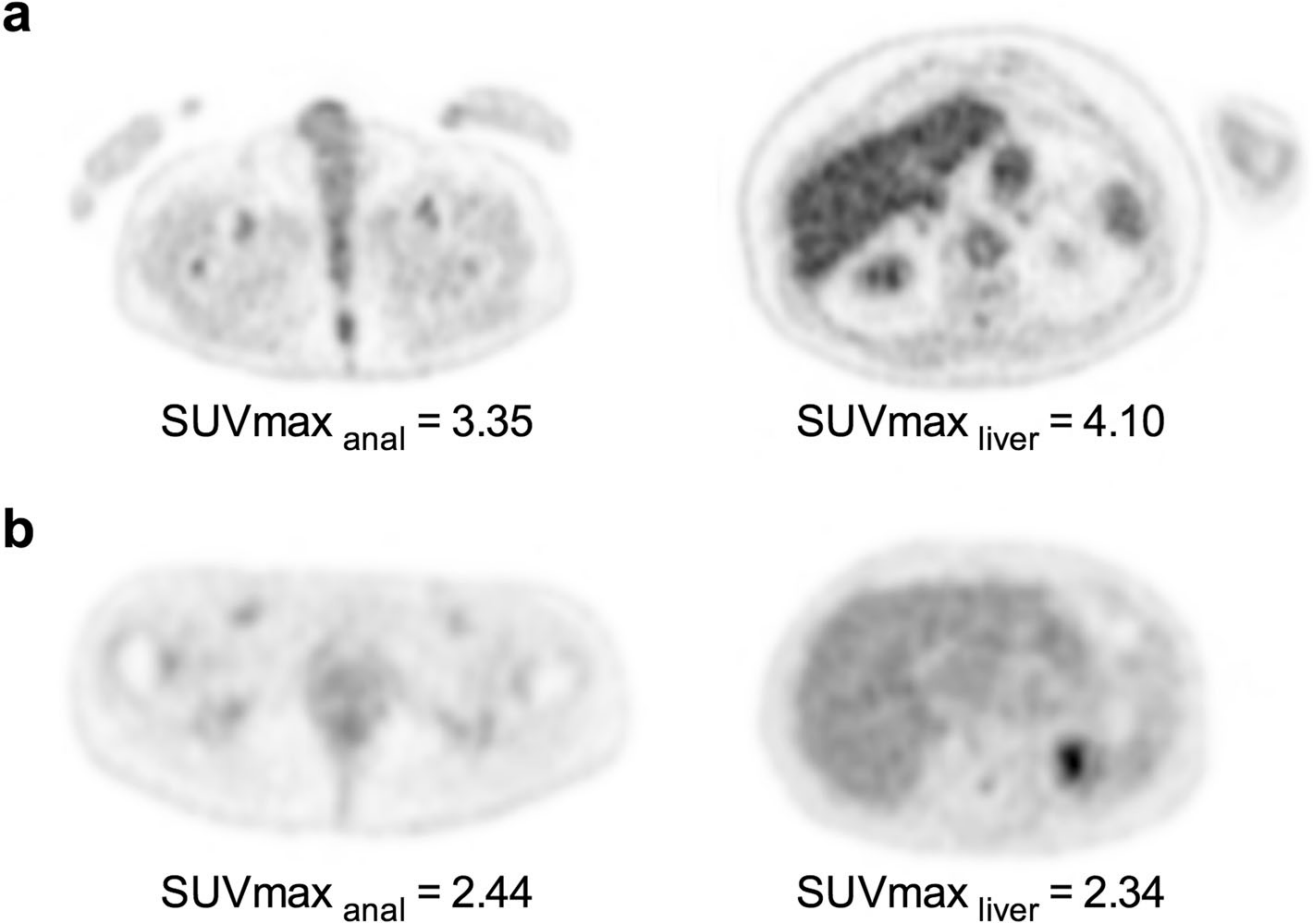
Axial EARL-accredited images centered on the anal canal (left panel) and the liver (right panel) of a patient classified with significant anal canal hypermetabolism on visual analysis who would be classified “negative” using liver uptake as reference (**a**) and a patient classified with basal anal canal hypermetabolism who would be classified as “positive” using liver uptake as reference (**b**)

Notable strengths of the study are the important number of patients included, as well as the high rate of entirely completed questionnaires (89%) and the use of EARL-compliant SUVs for the sake of pooling quantitative data from two systems (Siemens analogic and Phillips digital systems) and being able to export our results to other EARL accredited centers. However, we underwent both visual and quantitative analysis of anal canal uptakes. Visual assessment led to a strong agreement between observers but the use of a quantitative threshold to identify patients with a significant anal canal uptake hypermetabolism gave better results. Using a SUV_max___EARL_ cutoff value superior to 2.96 led to an almost perfect agreement between observers (κ = 0.88), thus demonstrating that our results could be easily extrapolated to other EARL-accredited PET centers. The use of an organ of reference, in particular the physiological uptake of the liver, to determine the presence of a significant anal canal uptake was not retained because of the high inter-individual variability. For example, in the random dataset of 100 patients attributed to the second observer, the liver uptake ranged from 1.89 to 4.38 with a median value equal to 2.95. Indeed, it is well-known that liver uptake can be influenced by various factors as BMI [18] steatosis [19], as well as the blood glucose level [20] and time interval between ^18^F-FDG injection [21] and PET acquisition [22]. Therefore, there is a significant risk to overestimate the event in patients with low liver uptake and underestimate the event in those with high liver uptake, whatever the underlying cause. Representative examples of these two scenarios are presented in Fig. 5. Of note, previous studies dealing with post-treatment PET examinations of anal cancer patients did not use quantitative data (nor SUV threshold or organ of reference) and were only based on visual assessment [3, 23, 24].

Concerning the limitations of the present study, we can first notice the risk of bias linked to the data collection method. For example, the antecedent of anal condyloma could not be evaluated because most of the patients actually did not have a good knowledge of what this disease is. However, rare cases of hypermetabolic condylomas had been described in voluminous lesions that could be encountered in immunodeficient patients as HIV-infected ones [25]. As none of the patients was concerned by this clinical situation in the present study, we can argue that it is unlikely to be a confounding factor. Moreover, concerning the question of a history of an anal invasive act (including sexual intercourse), even in the absence of the investigator in the room during the questionnaire filling process, there was surely a social desirability bias due to the nature of the question [26]. We could then hypothesize that the frequency of anal invasive act and its impact on anal canal hypermetabolism were underestimated. Secondly, the purpose of this study was to pave the way for more specific interpretation of post radio-chemotherapy PET studies in anal cancer patients. However, the recruitment of our PET center did not allow us to conduct an analysis of this specific sub-population. Indeed, in this 2-month inclusion period, only 3 patients had histories of anal cancer. Therefore, further works are needed to ensure that the present results could be extrapolated in this context.

## Conclusions

According to our study, the predictive factors of physiological anal canal hypermetabolism are high BMI, male gender, history of hemorrhoid, and rectum filled with only feces. Presently, an anal canal SUV_max___EARL_ superior to 2.96 seems to be an efficient threshold to identify patients with a significant anal canal hypermetabolism. This may pave the way to a more specific interpretation of post radio-chemotherapy PET evaluations of anal canal cancer and to the optimization of protocols, provided that these findings are validated in this specific population.

## Data Availability

Data from this study are available upon reasonable request.

## Abbreviations

EANM: European Association of Nuclear Medicine
EARL: EANM Research Ltd
FDG: Fluor-18 fluorodeoxyglucose
MIP: Maximum intensity projection
OSEM: Ordered subset expectation maximization
PET: Positron emission tomography
PSF: Point spread function
SUV: Standardized uptake value
SUV_max_: Maximum SUV
SUV_peak_: Peak SUV
